# Drug Interaction Between Erlotinib and Itraconazole in a Patient With Non–Small Cell Lung Cancer

**DOI:** 10.1155/crom/7788444

**Published:** 2026-04-07

**Authors:** Hayato Yokota, Satoshi Goshima, Yuji Okuda, Sho Sakamoto, Masahide Takeda, Kazuhiro Sato, Yumiko Akamine, Katsutoshi Nakayama, Masatomo Miura

**Affiliations:** ^1^ Department of Pharmacy, Akita University Hospital, Akita, Japan, akita-u.ac.jp; ^2^ Department of Internal Medicine, Division of Respiratory Medicine, Akita University School of Medicine, Akita, Japan, akita-u.ac.jp; ^3^ Department of Pharmacokinetics, Akita University Graduate School of Medicine, Akita, Japan, akita-u.ac.jp

**Keywords:** drug–drug interaction, erlotinib, itraconazole, non–small cell lung cancer, trough plasma concentration

## Abstract

**Introduction:**

Erlotinib (ERL) is metabolized primarily by cytochrome P450 (CYP) 3A4. We report a case of non–small cell lung cancer in which plasma ERL concentrations increased following coadministration of itraconazole (ITCZ).

**Case Presentation:**

In this patient, a 67‐year‐old man receiving ERL 150 mg/day, the trough plasma concentration (*C*_0_) of ERL and its major metabolite, O‐desmethyl ERL (OSI‐420), increased following coadministration of ITCZ. The total clearance of ERL at steady state was 6.1 L/h. The mean *C*_0_ of ERL and OSI‐420 were 437 and 47.1 ng/mL, respectively, and the *C*_0_ ratio of OSI‐420/ERL was 0.108. With coadministration of oral ITCZ (200 mg/day capsule), the mean *C*_0_ of ERL and OSI‐420 increased to 1124 and 166 ng/mL, respectively, and the mean *C*_0_ ratio of OSI‐420/ERL increased to 0.147. The mean *C*_0_ of ITCZ and the sum of ITCZ and the active metabolite hydroxyitraconazole (OH‐ITCZ) were 109 and 271 ng/mL, respectively. The mean *C*_0_ of ERL increased approximately 2.5‐fold.

**Conclusion:**

Even at the relatively lower *C*_0_ of ITCZ (compared with the recommended target value), ITCZ coadministration increased the *C*_0_ of ERL. In patients with a sufficient ERL *C*_0_ of more than 500 ng/mL prior to ITCZ coadministration or patients exhibiting adequate absorption of oral ITCZ, the risk of ERL‐related adverse events with ITCZ coadministration may increase due to further elevation of the ERL *C*_0_. Therefore, when ERL is coadministered with ITCZ, careful monitoring for adverse effects and appropriate dose adjustments are required, considering potential changes in ERL concentrations. Management using the *C*_0_ of ERL and ITCZ may be necessary.

## 1. Introduction

Erlotinib (ERL), a selective epidermal growth factor receptor (EGFR) tyrosine kinase inhibitor, is a standard treatment for patients with *EGFR* mutation‐positive non–small cell lung cancer (NSCLC) [1–3]. Combination therapy with ERL and ramucirumab significantly prolonged progression‐free survival compared with ERL plus placebo in patients with common *EGFR* mutations, including Exon 19 deletions and the Exon 21 L858R mutation [4]. ERL is demethylated primarily by cytochrome P450 (CYP) 3A4 and minorly by CYP3A5 and CYP1A2 to the major metabolite, O‐desmethyl ERL (OSI‐420); subsequently, OSI‐420 is oxidized to a carboxylic acid [5–7]. Ketoconazole has been reported to increase the area under the plasma concentration‐time curve (AUC) of ERL by 67% in healthy subjects [8]. Therefore, the Food and Drug Administration recommends dose reduction or avoidance of ERL coadministration with potent CYP3A4 inhibitors whenever possible [8].

Itraconazole (ITCZ) is widely used to prevent and treat systemic fungal infections, including aspergillosis [9]; however, to date, no reports have examined the drug interaction between ERL and ITCZ in patients with lung cancer. Here, we report on a patient with lung adenocarcinoma who was taking ERL coadministered with ITCZ.

## 2. Case Presentation

**Figure 1:**
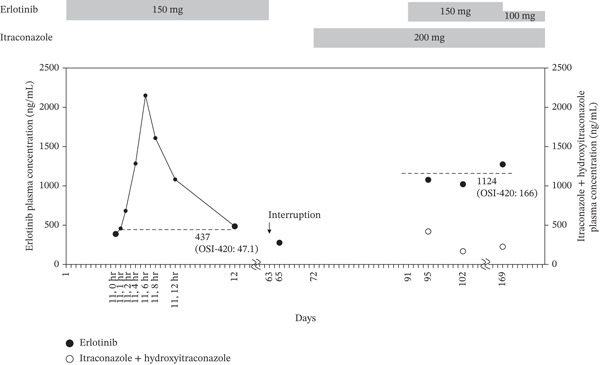
Plasma concentrations of erlotinib during treatment. Black circles represent the trough plasma concentration (large circles) and postdose plasma concentrations (small circles) of erlotinib during treatment. White circles represent total plasma concentrations of itraconazole and its active metabolite, hydroxyitraconazole.

A 67‐year‐old man was diagnosed with Stage IIIC pulmonary adenocarcinoma of the right upper lobe. He had a history of dermatomyositis associated with antisynthetase syndrome (anti‐Jo‐1 antibody subtype), for which he had been receiving prednisolone (PSL: 40 mg/day). Genetic testing revealed an *EGFR* Exon 21 L858R mutation. The patient was started on combination therapy with oral ERL 150 mg/day and intravenous ramucirumab 580 mg (10 mg/kg) every 2 weeks. The AUC_0-24_ and the total clearance of ERL in this patient were 24,732 ng·h/mL and 6.1 L/h, respectively. The mean trough plasma concentrations (*C*_0_) of ERL and OSI‐420 were 437 and 47.1 ng/mL, respectively, and the *C*_0_ ratio of OSI‐420/ERL was 0.108 (Figure 1). ERL was well tolerated, with only dermatitis acneiform of Grade 1 per the Common Terminology Criteria for Adverse Events Version 5.0. observed on Day 33 after initiation. Computed tomography (CT) scans demonstrated a decrease in the size of the right upper lobe mass.

The patient developed shortness of breath 2 months after the initiation of the ERL plus ramucirumab combination, when the dose of PSL was tapered from 40 to 10 mg/day. Exacerbation of interstitial lung disease (ILD) associated with dermatomyositis was suspected. CT revealed patchy consolidations accompanied by reticular opacities along the bronchovascular bundles in the subpleural regions, consistent with prior ILD findings. ERL was discontinued temporarily on Day 63 after therapy initiation. Because the patient’s serum creatine kinase (CK) was elevated to 888 U/L on Day 64, the PSL dose was increased to 65 mg/day. Two days (48 h) after ERL discontinuation (Day 65), the plasma concentration of ERL and OSI‐420 decreased to 276 and 32.8 ng/mL, respectively, and the dermatitis acneiform was resolved.

Laboratory tests showed a higher *β*‐D‐glucan level of 69.5 pg/mL, raising suspicion of fungal infection. As a result, treatment with oral ITCZ 200 mg/day was started on Day 72. Although the *β*‐D‐glucan level 2 days after initiation of ITCZ was 44.4 pg/mL, it decreased to 33.2 pg/mL on Day 10 after initiation of ITCZ therapy. In response to a decrease in CK levels to 200 U/L, the PSL dose was reduced to 50 mg/day. Because it was judged that ERL was not a cause of the ILD exacerbation, without a change in ITCZ therapy, ERL was restarted at 150 mg/day on Day 91.

On Day 95, the *C*_0_ of ERL and OSI‐420 with ITCZ were 1077 and 154 ng/mL, respectively, whereas the *C*_0_ of ITCZ and the sum of ITCZ and the active metabolite hydroxyitraconazole (OH‐ITCZ) were 181 and 419 ng/mL, respectively. On Day 102, the *C*_0_ of ERL and OSI‐420 with ITCZ were 1021 and 140 ng/mL, respectively, and the *C*_0_ of ITCZ and the sum of ITCZ and OH‐ITCZ were 76 and 168 ng/mL, respectively (Figure 1). The PSL dose was tapered gradually to 20 mg/day, and the CK level normalized to 39 U/L, allowing the patient to be discharged.

On Day 127, the patient developed a recurrence of dermatitis acneiform related to ERL, and a topical steroid was prescribed. However, the dermatitis acneiform worsened to Grade 3 6 weeks later (Day 169), and the *C*_0_ of ERL and OSI‐420 with ITCZ on the same day were 1273 and 203 ng/mL, respectively. Because of insufficient improvement, the ERL dose on Day 169 was reduced from 150 to 100 mg/day, and treatment was continued. The mean *C*_0_ of ERL increased approximately 2.5‐fold, from 437 to 1124 ng/mL, with coadministration of ITCZ, whereas the mean OSI‐420 *C*_0_ increased from 47.1 to 166 ng/mL with coadministration of ITCZ, and the *C*_0_ ratio OSI‐420/ERL increased from 0.108 to 0.147. On the same day, the *C*_0_ of ITCZ and the sum of ITCZ and OH‐ITCZ were 71 and 225 ng/mL, respectively. The patient, who was immunosuppressed due to PSL therapy, continued to receive ITCZ, during which no clinical signs of fungal infection were observed. The patient remains on therapy with ERL 100 mg/day plus ramucirumab.

## 3. Discussion

In this case, increased concentrations of ERL and its active metabolite, OSI‐420, were observed after initiation of concomitant treatment with ITCZ in a patient with NSCLC. To our knowledge, this is the first case report to measure the plasma concentrations of ITCZ, ERL, and OSI‐420 to assess drug interactions between ITCZ and ERL. In the presence of ITCZ, the *C*_0_ ratio of OSI‐420/ERL increased from 0.108 to 0.147, approximately a 1.4‐fold increase. Similar studies have demonstrated that CYP3A4 inhibitors, such as ketoconazole and BAS‐100, increase the AUC of OSI‐420 [7, 10]. This may suggest that ERL is metabolized by CYP1A2 in the presence of inhibition of CYP3A4/5 by ITCZ. In addition, CYP3A4 may be involved in the metabolic pathway that converts OSI‐420 to carboxylic acid.

A target ERL *C*_0_ of 500 ng/mL in humans has been reported to be adequate to achieve EGFR inhibition; therefore, this target *C*_0_ is widely referenced [11]. During ERL therapy in this patient, the mean ERL *C*_0_ was 437 ng/mL, which was lower than the target *C*_0_ of 500 ng/mL. However, since tumor shrinkage was observed in the early phase of treatment, the relationship between ERL *C*_0_ and tumor response during combination therapy with ramucirumab should be interpreted with caution. The target *C*_0_ of ERL with ramucirumab may be different from that with ERL alone. In five previous clinical studies, the mean ERL *C*_0_ at steady state with administration of ERL 150 mg/day ranged from 795 to 1642 ng/mL [12–16]. In the current case, following ITCZ administration, the mean ERL *C*_0_ increased to 1124 ng/mL, which was fortunately in the average *C*_0_ range after administration of ERL 150 mg/day. In this patient, the clearance of ERL with coadministration of ITCZ was reduced by 2.5‐fold; consequently, the risk of severe adverse events was increased. The total clearance of ERL without ITCZ was 6.1 L/h; however, with ITCZ administration, it changed to about 2.4 L/h. Therefore, careful monitoring after combination therapy with ITCZ is necessary.

Dermatitis acneiform is a common adverse effect of ERL, with reported incidence rates of 73%–82.5% during monotherapy [1–3]. In the RELAY trial of ERL with ramucirumab, the incidence of dermatitis acneiform of Grades 1–2 and Grade 3 was 52% and 15%, respectively [4]. No tumor progression was observed during the coadministration of ERL and ITCZ, and we felt that these drugs could be used in combination, as long as adverse effects remained tolerable. In this case, although ERL was well tolerated during 2 months of ITCZ coadministration, the patient developed dermatitis acneiform. Patients with skin toxicity of Grade 3 were reported to have a significantly higher ERL *C*_0_, with a cutoff of 1810 ng/mL, compared with those with skin toxicity of Grades 0–2 (2690 vs. 1210 ng/mL, *p* = 0.004) [14]. In our case, the ERL *C*_0_ with dermatitis acneiform of Grade 3 was 1124 ng/mL, an average *C*_0_. Therefore, it may be difficult to predict skin toxicity based on the ERL *C*_0_.

A *C*_0_ of ITCZ of more than 500 ng/mL or a sum of *C*_0_ of ITCZ and OH‐ITCZ of more than 1000 ng/mL is generally recommended for treatment of *Aspergillus* species infections [17, 18]. Although the sum of the *C*_0_ of ITCZ and OH‐ITCZ in this patient was below that threshold, the administration of ITCZ 200 mg/day rapidly decreased *β*‐D‐glucan levels. Therefore, therapy using the same dose was continued. The degree of drug interaction between ERL and ITCZ might be large when the *C*_0_ of ITCZ is higher than the target *C*_0_ of 500 ng/mL, because ITCZ inhibits CYP3A4 in a dose‐dependent manner [19].

## 4. Conclusion

Even at a relatively lower *C*_0_ of ITCZ (compared with the recommended target value), ITCZ coadministration increased the *C*_0_ of ERL. In patients with an ERL *C*_0_ that is more than 500 ng/mL prior to coadministration of ITCZ or patients exhibiting an adequate absorption of oral ITCZ, the risk of adverse events with ERL with coadministration of ITCZ may increase due to further elevation of the ERL *C*_0_. Therefore, when ERL is coadministered with ITCZ, careful monitoring for adverse effects and appropriate dose adjustments are required, taking into account potential changes in ERL concentrations. Management using the *C*_0_ of ERL and ITCZ may be helpful in selected patients.

**Nomenclature**
AUC: area under the plasma concentration‐time curve
*C*_0_
: trough plasma concentration
CK: creatine kinase
CT: computed tomography
CYP: cytochrome P450
EGFR: epidermal growth factor receptor
ERL: erlotinib
ILD: interstitial lung disease
ITCZ: itraconazole
NSCLC: non–small cell lung cancer
OH‐ITCZ: hydroxyitraconazole
PSL: prednisolone

## Funding

The study was funded by the Japan Society for the Promotion of Science (10.13039/501100001691, 25H00265).

## Ethics Statement

The study was conducted according to the principles of the Declaration of Helsinki. The study protocol was approved by the Ethics Committee of Akita University School of Medicine (Approval Number 2826).

## Consent

Written informed consent for publication was obtained from the patient described in this article.

## Conflicts of Interest

The authors declare no conflicts of interest.

## Data Availability

The data that support the findings of this study are available from the corresponding author upon reasonable request.

## References

[1] Zhou C. , Wu Y. L. , Chen G. , Feng J. , Liu X. Q. , Wang C. , Zhang S. , Wang J. , Zhou S. , Ren S. , Lu S. , Zhang L. , Hu C. , Hu C. , Luo Y. , Chen L. , Ye M. , Huang J. , Zhi X. , Zhang Y. , Xiu Q. , Ma J. , Zhang L. , and You C. , Erlotinib Versus Chemotherapy as First-Line Treatment for Patients With Advanced EGFR Mutation-Positive Non-Small-Cell Lung Cancer (OPTIMAL, CTONG-0802): A Multicentre, Open-Label, Randomised, Phase 3 Study, Lancet Oncology. (2011) 12, no. 8, 735–742, 10.1016/S1470-2045(11)70184-X, 2-s2.0-79960889662, 21783417. 21783417

[2] Rosell R. , Carcereny E. , Gervais R. , Vergnenegre A. , Massuti B. , Felip E. , Palmero R. , Garcia-Gomez R. , Pallares C. , Sanchez J. M. , Porta R. , Cobo M. , Garrido P. , Longo F. , Moran T. , Insa A. , de Marinis F. , Corre R. , Bover I. , Illiano A. , Dansin E. , de Castro J. , Milella M. , Reguart N. , Altavilla G. , Jimenez U. , Provencio M. , Moreno M. A. , Terrasa J. , Muñoz-Langa J. , Valdivia J. , Isla D. , Domine M. , Molinier O. , Mazieres J. , Baize N. , Garcia-Campelo R. , Robinet G. , Rodriguez-Abreu D. , Lopez-Vivanco G. , Gebbia V. , Ferrera-Delgado L. , Bombaron P. , Bernabe R. , Bearz A. , Artal A. , Cortesi E. , Rolfo C. , Sanchez-Ronco M. , Drozdowskyj A. , Queralt C. , de Aguirre I. , Ramirez J. L. , Sanchez J. J. , Molina M. A. , Taron M. , Paz-Ares L. , and Spanish Lung Cancer Group in collaboration with Groupe Français de Pneumo-Cancérologie and Associazione Italiana Oncologia Toracica , Erlotinib Versus Standard Chemotherapy as First-Line Treatment for European Patients With Advanced EGFR Mutation-Positive Non-Small-Cell Lung Cancer (EURTAC): A Multicentre, Open-Label, Randomised Phase 3 Trial, Lancet Oncology. (2012) 13, no. 3, 239–246, 10.1016/S1470-2045(11)70393-X, 2-s2.0-84857502654, 22285168. 22285168

[3] Yamamoto N. , Goto K. , Nishio M. , Chikamori K. , Hida T. , Maemondo M. , Katakami N. , Kozuki T. , Yoshioka H. , Seto T. , Tajima K. , and Tamura T. , Final Overall Survival in JO22903, a Phase II, Open-Label Study of First-Line Erlotinib for Japanese Patients With EGFR Mutation-Positive Non-Small-Cell Lung Cancer, International Journal of Oncology. (2017) 22, no. 1, 70–78, 10.1007/s10147-016-1039-0, 2-s2.0-85014101309, 27659294. PMC5306267 27659294

[4] Nakagawa K. , Garon E. B. , Seto T. , Nishio M. , Ponce Aix S. , Paz-Ares L. , Chiu C. H. , Park K. , Novello S. , Nadal E. , Imamura F. , Yoh K. , Shih J. Y. , Au K. H. , Moro-Sibilot D. , Enatsu S. , Zimmermann A. , Frimodt-Moller B. , Visseren-Grul C. , Reck M. , Chu Q. , Cortot A. , Pujol J. L. , Moro-Sibilot D. , Fabre E. , Lamour C. , Bischoff H. , Kollmeier J. , Reck M. , Kimmich M. , Engel-Riedel W. , Hammerschmidt S. , Schütte W. , Syrigos K. , Ho J. C. M. , Au K. H. , Novello S. , Ardizzoni A. , Pasello G. , Gregorc V. , del Conte A. , Galetta D. , Takahashi T. , Nakagawa K. , Nishio M. , Yoh K. , Seto T. , Imamura F. , Kumagai T. , Hotta K. , Goto Y. , Hosomi Y. , Sakai H. , Takiguchi Y. , Kim Y. H. , Kurata T. , Yamaguchi H. , Daga H. , Okamoto I. , Satouchi M. , Ikeda S. , Kasahara K. , Atagi S. , Azuma K. , Kumagai T. , Aoe K. , Kumagai T. , Aoe K. , Horio Y. , Yamamoto N. , Tanaka H. , Watanabe S. , Nogami N. , Ozaki T. , Koyama R. , Hirashima T. , Kaneda H. , Tomii K. , Fujita Y. , Seike M. , Nishimura N. , Kato T. , Ichiki M. , Saka H. , Hirano K. , Nakahara Y. , Sugawara S. , Park K. , Kim S. W. , Min Y. J. , Lee H. W. , Kang J. H. , An H. J. , Lee K. H. , Kim J. S. , Lee G. W. , Lee S. Y. , Alexandru A. , Udrea A. A. , Juan-Vidal Ó. , Nadal-Alforja E. , Gil-Bazo I. , Ponce-Aix S. , Paz-Ares L. , Rubio-Viqueira B. , Alonso Garcia M. , Felip Font E. , Fuentes Pradera J. , Coves Sarto J. , Lin M. C. , Su W. C. , Hsia T. C. , Chang G. C. , Wei Y. F. , Chiu C. H. , Shih J. Y. , Su J. , Cicin I. , Goksel T. , Harputluoglu H. , Ozyilkan O. , Henning I. , Popat S. , Hatcher O. , Mileham K. , Acoba J. , Garon E. , Jung G. , Raj M. , Martin W. , and Dakhil S. , Ramucirumab Plus Erlotinib in Patients With Untreated, EGFR-Mutated, Advanced Non-Small-Cell Lung Cancer (RELAY): A Randomised, Double-Blind, Placebo-Controlled, Phase 3 Trial, Lancet Oncology. (2019) 20, no. 12, 1655–1669, 10.1016/S1470-2045(19)30634-5, 31591063. 31591063

[5] Li J. , Zhao M. , He P. , Hidalgo M. , and Baker S. D. , Differential Metabolism of Gefitinib and Erlotinib by Human Cytochrome P 450 Enzymes, Clinical Cancer Research. (2007) 13, no. 12, 3731–3737, 10.1158/1078-0432.CCR-07-0088, 2-s2.0-34250694236, 17575239. 17575239

[6] Ling J. , Johnson K. A. , Miao Z. , Rakhit A. , Pantze M. P. , Hamilton M. , Lum B. L. , and Prakash C. , Metabolism and Excretion of Erlotinib, a Small Molecule Inhibitor of Epidermal Growth Factor Receptor Tyrosine Kinase, in Healthy Male Volunteers, Drug Metabolism and Disposition. (2006) 34, no. 3, 420–426, 10.1124/dmd.105.007765, 2-s2.0-33344461165, 16381666. 16381666

[7] Rakhit A. , Pantze M. P. , Fettner S. , Jones H. M. , Charoin J. E. , Riek M. , Lum B. L. , and Hamilton M. , The Effects of CYP3A4 Inhibition on Erlotinib Pharmacokinetics: Computer-Based Simulation (SimCYP) Predicts In Vivo Metabolic Inhibition, European Journal of Pharmacology. (2008) 64, no. 1, 31–41, 10.1007/s00228-007-0396-z, 2-s2.0-38049054403, 18000659. 18000659

[8] U.S. Food and Drug Administration. TARCEVA (Erlotinib) Tablets, for Oral Use, 2016, https://www.fda.gov/drugsatfda.

[9] Walsh T. J. , Anaissie E. J. , Denning D. W. , Herbrecht R. , Kontoyiannis D. P. , Marr K. A. , Morrison V. A. , Segal B. H. , Steinbach W. J. , Stevens D. A. , van Burik J. , Wingard J. R. , Patterson T. F. , and Infectious Diseases Society of America , Treatment of Aspergillosis: Clinical Practice Guidelines of the Infectious Diseases Society of America, Clinical Infectious Diseases. (2008) 46, no. 3, 327–360, 10.1086/525258, 2-s2.0-39449095735, 18177225. 18177225

[10] Smith N. F. , Baker S. D. , Gonzalez F. J. , Harris J. W. , Figg W. D. , and Sparreboom A. , Modulation of Erlotinib Pharmacokinetics in Mice by a Novel Cytochrome P 450 3A4 Inhibitor, BAS 100, British Journal of Cancer. (2008) 98, no. 10, 1630–1632, 10.1038/sj.bjc.6604353, 2-s2.0-43649104767, 18475295. 18475295 PMC2391127

[11] Verheijen R. B. , Yu H. , Schellens J. H. M. , Beijnen J. H. , Steeghs N. , and Huitema A. D. R. , Practical Recommendations for Therapeutic Drug Monitoring of Kinase Inhibitors in Oncology, Clinical Pharmacology and Therapeutics. (2017) 102, no. 5, 765–776, 10.1002/cpt.787, 2-s2.0-85028910046, 28699160. 28699160 PMC5656880

[12] Yamamoto N. , Horiike A. , Fujisaka Y. , Murakami H. , Shimoyama T. , Yamada Y. , and Tamura T. , Phase I Dose-Finding and Pharmacokinetic Study of the Oral Epidermal Growth Factor Receptor Tyrosine Kinase Inhibitor Ro50-8231 (Erlotinib) in Japanese Patients With Solid Tumors, Cancer Chemotherapy and Pharmacology. (2008) 61, no. 3, 489–496, 10.1007/s00280-007-0494-8, 2-s2.0-37249041552, 17483950. 17483950

[13] Hidalgo M. , Siu L. L. , Nemunaitis J. , Rizzo J. , Hammond L. A. , Takimoto C. , Eckhardt S. G. , Tolcher A. , Britten C. D. , Denis L. , Ferrante K. , von Hoff D. D. , Silberman S. , and Rowinsky E. K. , Phase I and Pharmacologic Study of OSI-774, an Epidermal Growth Factor Receptor Tyrosine Kinase Inhibitor, in Patients With Advanced Solid Malignancies, Journal of Clinical Oncology. (2001) 19, no. 13, 3267–3279, 10.1200/JCO.2001.19.13.3267, 2-s2.0-0035398631, 11432895. 11432895

[14] Tiseo M. , Andreoli R. , Gelsomino F. , Mozzoni P. , Azzoni C. , Bartolotti M. , Bortesi B. , Goldoni M. , Silini E. M. , de Palma G. , Mutti A. , and Ardizzoni A. , Correlation Between Erlotinib Pharmacokinetics, Cutaneous Toxicity and Clinical Outcomes in Patients With Advanced Non-Small Cell Lung Cancer (NSCLC), Lung Cancer.(2014) 83, no. 2, 265–271, 10.1016/j.lungcan.2013.12.001, 2-s2.0-84892893614, 24388705. 24388705

[15] Liao D. , Yao D. , Liu N. , Cao L. , Xiang D. , Yang N. , Zhang Y. , Jiang W. , and Zhou C. , Correlation of Plasma Erlotinib Trough Concentration With Skin Rash in Chinese NSCLC Patients Harboring Exon 19 Deletion Mutation, Cancer Chemotherapy and Pharmacology. (2018) 82, no. 3, 551–559, 10.1007/s00280-018-3642-4, 2-s2.0-85050557877, 30039303. 30039303

[16] Steffens M. , Paul T. , Hichert V. , Scholl C. , von Mallek D. , Stelzer C. , Sörgel F. , Reiser B. , Schumann C. , Rüdiger S. , Boeck S. , Heinemann V. , Kächele V. , Seufferlein T. , and Stingl J. , Dosing to Rash? The Role of Erlotinib Metabolic Ratio From Patient Serum in the Search of Predictive Biomarkers for EGFR Inhibitor-Mediated Skin Rash, European Journal of Cancer Care. (2016) 55, 131–139, 10.1016/j.ejca.2015.11.022, 2-s2.0-84955247671, 26820683. 26820683

[17] Nakamura Y. , Matsumoto K. , Sato A. , and Morita K. , Effective Plasma Concentrations of Itraconazole and Its Active Metabolite for the Treatment of Pulmonary Aspergillosis, Journal of Infection and Chemotherapy. (2020) 26, no. 2, 170–174, 10.1016/j.jiac.2019.08.002, 2-s2.0-85071468818, 31481305. 31481305

[18] McCreary E. K. , Davis M. R. , Narayanan N. , Andes D. R. , Cattaneo D. , Christian R. , Lewis R. E. , Watt K. M. , Wiederhold N. P. , and Johnson M. D. , Utility of Triazole Antifungal Therapeutic Drug Monitoring: Insights From the Society of Infectious Diseases Pharmacists, Pharmacotherapy. (2023) 43, no. 10, 1043–1050, 10.1002/phar.2850. 37459118

[19] Templeton I. , Peng C. C. , Thummel K. E. , Davis C. , Kunze K. L. , and Isoherranen N. , Accurate Prediction of Dose-Dependent CYP3A4 Inhibition by Itraconazole and Its Metabolites From In Vitro Inhibition Data, Clinical Pharmacology and Therapeutics. (2010) 88, no. 4, 499–505, 10.1038/clpt.2010.119, 2-s2.0-77957019850, 20739919. 20739919 PMC3056523

